# Joint effects of prenatal PM_2.5_ and heavy metals on preterm birth: evidence from a nested case–control study in China

**DOI:** 10.3389/fpubh.2025.1701012

**Published:** 2026-01-21

**Authors:** Jinxiu Feng, Ziyi He, Xin Ming, Yunping Yang, Yannan Li, Dong Ting, Xinzhen Chen, Min Zhang, Yankun Wang, Li Zhou, Wenzheng Zhou

**Affiliations:** 1 Women and Children's Hospital of Chongqing Medical University, China; 2 Chongqing Health Center for Women and Children, China; 3 Chongqing Research Center for Prevention & Control of Maternal and Child Disease and Public Health, Chongqing, China; 4 Department of Epidemiology, School of Public Health, Chongqing Medical University, Chongqing, China

**Keywords:** preterm birth, heavy metals, fine particulate matter, maternal exposure, mixture modeling, environmental epidemiology

## Abstract

**Background:**

Preterm birth (PTB) is a global health concern. The combined effects of prenatal PM_2.5_ and heavy metal exposures on PTB risk remain unclear.

**Methods:**

A nested case-control study included 111 PTB cases and 124 controls from Chongqing. Maternal blood concentrations of 18 trace elements were measured in mid-pregnancy. Fine particulate matter (PM_2.5_) exposure data were obtained from the Tracking Air Pollution in China (TAP). Residential addresses were geocoded and linked to these exposure estimates. Logistic regression, weighted quantile sum (WQS), grouped WQS (GWQS), and bayesian kernel machine regression (BKMR) models were used to evaluate individual, mixture and interaction effects.

**Results:**

Higher PM_2.5_ exposure during the second trimester and early-to-mid pregnancy was associated with increased PTB risk. Cadmium, selenium, and zinc were positively associated with PTB, whereas lithium and nickel were inversely associated. WQS models indicated the mixture was associated with elevated PTB risk (OR: 2.01, 95% CI: 1.09, 3.71), with PM_2.5_ contributing the largest weight, lithium and nickel were inversely associated (OR: 0.36, 95% CI: 0.18, 0.74). GWQS models showed that PM_2.5_ (OR: 2.24, 95% CI: 1.68, 3.07) and essential trace elements (ETE) (OR: 2.32, 95% CI: 1.60, 3.44) positively associated with PTB, whereas conditionally essential trace elements (CTE) were inversely associated (OR: 0.37, 95% CI: 0.23, 0.59). Seven-group GWQS analyses suggested potential interaction between PM_2.5_ and ETE (OR: 4.44, 95% CI: 2.33, 9.10) and inverse associations with CTE. BKMR revealed nonlinear mixture effects and a non-monotonic effect of lithium.

**Conclusion:**

Pregnancy exposure to PM_2.5_ and heavy metal mixtures were associated PTB risk, with evidence of complex and potentially interactive associations. These findings highlight the importance of integrated assessments of multiple environmental exposures and targeted public health strategies to reduce maternal and child health risks.

## 1 Introduction

Preterm birth (PTB), defined as a live birth before 37 completed weeks of gestation (1), is a major global public health challenge. In 2020, an estimated 13.4 million infants were born preterm, representing approximately 10.6% of all live births worldwide (2, 3). According to recent estimates from the Global Burden of Disease (GBD) study, the incidence of PTB has increased by 43.1% between 1990 and 2019 in countries with a low sociodemographic index (SDI), including many developing nations (4). Developing countries, including China, are disproportionately affected, emphasizing the need for comprehensive policies and preventive measures to address this issue effectively.

Globally, PTB is the leading cause of mortality among children under five. In 2019, complications related to PTB accounted for 17.7% of all deaths in this age group, with approximately 900,000 fatalities (5). In China, similar trends have been observed, where PTB and its complications are the second leading cause of death among children under five, contributing to 17% of all deaths in this demographic (6). These alarming statistics highlight the critical role of PTB as a predictor of infant mortality (7).

Beyond its immediate impact on neonatal survival, PTB poses long-term risks to children's health, often resulting in developmental delays and chronic conditions. Addressing PTB is particularly urgent in low-SDI areas, where the burden is most pronounced. However, the underlying causes of PTB are complex, involving interactions between genetic predispositions and environmental factors.

Environmental exposures during pregnancy have garnered increasing attention as contributors to PTB. Epidemiological studies have reported associations between PTB and exposure to heavy metals such as lead (Pb) (8, 9), cadmium (Cd) (10, 11), mercury (Hg) (12, 13), copper (Cu) (14–16), zinc (Zn) (17–21), lithium (Li) (22–24), manganese (Mn) (14, 25), nickel (Ni) (26), and Arsenic (As) (27).

Additionally, fine particulate matter (PM_2.5_) has been recognized as a major global environmental health concern. Prolonged exposure to PM_2.5_ has been linked to increased risks of cardiovascular diseases, respiratory illnesses, and lung cancer (28). Growing evidence also highlights the role of maternal exposure to ambient PM_2.5_ during pregnancy as a significant risk factor for PTB (29, 30). The mechanisms by which PM_2.5_ contributes to PTB are complex, involving inflammatory and oxidative stress responses, as well as disruptions to immune function, placental development, and fetal growth (31, 32).

However, findings from these studies are inconsistent, partly due to regional variations in exposure levels and the focus on single pollutants. Moreover, pregnant individuals are simultaneously exposed to complex mixtures of environmental pollutants and micronutrients, which may interact in synergistic or antagonistic ways. Yet, few studies have comprehensively assessed the joint and interactive effects of PM_2.5_ and multiple trace elements on PTB risk.

In this study, we hypothesized that exposure to a mixture of PM_2.5_ and trace elements during pregnancy is associated with increased risk of PTB, and that certain components may exert protective or antagonistic effects. To test this hypothesis, we conducted a nested case-control study in Chongqing, China, measuring maternal blood concentrations of 18 trace elements and estimating gestational PM_2.5_ exposure using high-resolution spatiotemporal models. We employed multiple statistical approaches, including logistic regression, weighted quantile sum (WQS) regression, grouped WQS (GWQS), extended GWQS with interaction terms, and bayesian kernel machine regression (BKMR), to evaluate both individual and mixture effects. Our aim was to identify key environmental contributors and interactions that influence PTB risk, and to provide mechanistic insights for future interventions.

## 2 Materials and methods

### 2.1 Study design and population

This nested case-control study was conducted as part of the “Southwest Regional Natural Population Birth Cohort” at the Women's Hospital of Chongqing Medical University from December 2018 to May 2022. There were recruited based on the following inclusion criteria: (a) age ≥18 years; (b) first obstetric examination before 14 weeks of pregnancy; (c) Permanent residents of Chongqing who reside in the same location as their place of origin; (d) absence of psychiatric or severe medical conditions; and (e) provision of informed consent. Participants were excluded if they (a) could not provide informed consent; (b) had a history of genitourinary or psychiatric illnesses; or (c) experienced three or more miscarriages or planned to relocate before delivery. Recruitment involved structured questionnaires and face-to-face interviews.

Gestational age (GA) was determined based on the last menstrual period (LMP) and confirmed by ultrasound examination. Cases were defined as women who delivered before 37 completed weeks of gestation, whereas controls were selected from women who delivered between 37 and 42 weeks of gestation. After excluding participants with stillbirths, abnormal deliveries, multiple gestations, or incomplete data, we identified cases and controls from pregnancies with gestational age < 37 weeks and ≥37 weeks, respectively. A 1:2.2 matching ratio was applied based on maternal age, body mass index (BMI), gravidity, parity, education level, employment status, and annual household income to minimize potential confounding. In total, 111 PTB cases and 124 matched controls were included in the final analysis. Ethical approval was obtained from the Medical Ethics Committee of Chongqing Maternal and Child Health Care Institute.

### 2.2 Demographic and gestational information

Sociodemographic and pregnancy-related data were collected through structured interviews conducted by trained staff. Variables included maternal age (years), height (cm), weight (kg), gestational age (weeks), number of prior pregnancies (0, ≥1), parity (0, ≥1), education level (high school or less, some college, college graduate, graduate degree or higher), occupation (yes or no), drinking (yes or no), smoking (yes or no), assisted reproductive technology (ART) (yes or no), family income (< 60,000, 60,000–99,999 yuan, 100,000–199,999 yuan, or ≥200,000 yuan), and mode of delivery (vaginal, cesarean section). Prepregnancy BMI was calculated using prepregnancy weight and height. Follow-up visits occurred during each trimester to collect health information, with 10 mL of peripheral blood drawn in the second trimester. Additional data, including fetal sex, birth weight, and delivery outcomes, were obtained from electronic medical records.

### 2.3 Elemental detection

Venous blood samples were collected from participants during the second trimester using EDTA anticoagulation tubes after an overnight fast. The samples were centrifuged within 24 h to separate plasma and hematocrits, which were then divided into portions and stored at −80°C for future analysis. For preparation, the samples were diluted with a solution containing 1% nitric acid, 0.01% Triton X-100, and 0.5% n-butanol.

Detection of metal elements was performed using inductively coupled plasma mass spectrometry (ICP-MS, Thermo Fisher, USA). The sample introduction system included a quartz concentric nebulizer, a vortex atomizer, and a three-channel peristaltic pump. Calibration was conducted using the online internal standard addition method, with pure argon gas as the collision gas and a high-sensitivity standard mode for detection. Before analysis, the instrument was tuned and calibrated with mass spectrometry tuning liquid to ensure accurate operation. The instrument's operating parameters were as follows: auxiliary gas flow rate of 0.80 L/min, nebulizer gas flow rate of 1.02 L/min, cooling gas flow rate of 14.0 L/min, 10 scans per sample, a dwell time of 0.02 s, three repeated measurements, a delivery time of 55 s, a washing time of 50 s, an ion flame horizontal position of −0.8 mm, and an ion flame vertical position of 0.71 mm.

Following preprocessing, 18 metal elements were analyzed and quantified: Aluminum (Al), Arsenic (As), Barium (Ba), Cadmium (Cd), Chromium (Cr), Cobalt (Co), Copper (Cu), Iron (Fe), Lead (Pb), Lithium (Li), Manganese (Mn), Nickel (Ni), Selenium (Se), Silver (Ag), Thallium (Tl), Vanadium (V), Zinc (Zn). The standard curves of the 18 metals had R^2^ values ranging from 0.999 to 1.000. All plasma metal concentrations were above the detection limit (LOD).

### 2.4 PM_2.5_ exposure assessment

Air pollution data were obtained from the Tracking Air Pollution in China (TAP) framework (http://tapdata.org.cn), which provides extensive spatial coverage. We used high-resolution PM_2.5_ estimates that integrated MAIAC satellite-derived aerosol optical depth (AOD) and land use variables including road networks, population distribution, impervious surfaces, and vegetation indices at a 10 km resolution. These estimates were generated by combining the Weather Research and Forecasting-Community Multiscale Air Quality (WRF-CMAQ) modeling system, ground monitoring data, machine learning algorithms, and multiple data sources (33).

Participants' residential addresses were geocoded into latitude and longitude coordinates using the Google Maps Application Programming Interface (API), and these were matched to the gridded PM_2.5_ and component data. Conception timing was estimated based on the last menstrual period (LMP). Daily concentrations were aggregated to calculate average exposure during specific windows, including the first trimester (weeks 1–13), the second trimester (weeks 14–28), and early-to-mid trimester (1–28 weeks). For clarity, PM_2.5__T1 refers to fine particulate matter < 2.5 μm during the first trimester, PM_2.5__T2 refers to fine particulate matter < 2.5 μm during the second trimester, and PM_2.5__T1 + T2 represents cumulative fine particulate matter < 2.5 μm across the first to middle trimester.

### 2.5 Statistical analysis

In the descriptive analysis of this study, continuous variables such as participants' age, gestational age, BMI, and neonatal height and weight were presented as mean ± standard deviation (SD). Differences between groups were analyzed using the two-independent-sample t-test. Categorical data, including maternal BMI, education level, occupation, annual family income, previous pregnancies, parity, mode of delivery, and fetal gender, were expressed as percentages or constituent ratios, with differences analyzed using the Chi-squared test. To compare pollutant concentrations between the PTB and non-PTB groups, the Mann–Whitney U test was applied. Due to the significant positive skewness in pollutant concentration distributions, natural logarithm (ln) transformations were performed before statistical analyses. Pearson's correlation analysis was used to examine relationships among pollutant levels.

Since the study's outcome variable was dichotomous, we employed multivariable logistic regression to estimate odds ratios (ORs) and 95% confidence intervals (CIs) for associations between PTB and individual exposures. Models included 18 metals (Ag, Al, As, Ba, Cd, Co, Cr, Cu, Fe, Li, Mn, Ni, Pb, Rb, Se, Tl, V, Zn) and trimester-specific PM_2.5_ (T1, T2) along with early-to-mid pregnancy PM_2.5_. Covariates were adjusted for occupation category, ART, parity, pregnancy history, education, income, age, BMI and mode of delivery. Restricted cubic splines (RCS) with 3 knots (at the 10th, 50th, and 90th percentiles) were used to evaluate potential nonlinear exposure-response relationships for each pollutant. To further address the risk of false-positive findings due to multiple comparisons, *P*-values from the unadjusted logistic regression models were adjusted using the Benjamini–Hochberg False Discovery Rate (FDR) method.

However, traditional logistic regression models often face challenges such as multicollinearity and inflated variance factors (34). To address these limitations and better assess the combined effects of PM_2.5_ and 18 metals across different pregnancy periods on PTB, the WQS regression method was employed. WQS regression is more sensitive and precise than traditional regression approaches, such as lasso, adaptive lasso, and elastic net, in identifying significant mixture risk factors (35). This method, a statistical methodology adopted to evaluate the influence of complex exposure mixtures on outcomes, creates a WQS index (ranging from 0 to 1) based on pollutant quartiles, allowing for the evaluation of overall environmental exposure effects and the contribution of each mixture component (36), minimizing the potential biases and omissions resulting from individual factor analysis, enhancing the sensitivity in identifying mixture risk factors, and providing a more comprehensive portrayal of the overall impact of the mixture. Such analyses have been employed in comparable research (37).

Despite its advantages, the WQS model assumes uniform exposure directions (all positive or all negative), which may not align with real-world scenarios. To address this limitation, the GWQS model was introduced. The GWQS model is an expansion of the traditional WQS approach, allowing exposures to be grouped such that each group may have different association sizes and directions (a comparison of the performance of the two models can be found in an analysis) (38), thereby compensating for the limitations of the WQS model. Moreover, the GWQS enables a more comprehensive consideration of combinations of multiple exposures through a weighted approach.

In addition, we further applied BKMR to evaluate the joint and individual effects of PM_2.5_ and trace elements on PTB risk. BKMR is a flexible semi-parametric modeling approach that estimates the overall mixture effect while accounting for potential nonlinearity and interactions among highly correlated exposures (39). Posterior inclusion probabilities (PIPs) were used to assess the relative importance of each exposure in the model. The overall mixture effect was evaluated across exposure percentiles, and univariate exposure-response functions were visualized while holding all other exposures at their median levels.

In this study, the 18 metallic elements were categorized based on bioavailability and WHO criteria (40). Elements were grouped into conditional trace elements (Li, Rb, V), essential trace elements (Cr, Co, Cu, Fe, Mn, Se, Zn) and toxic trace elements (Al, As, Ag, Ba, Cd, Ni, Pb, Ti). These groupings align with approaches used in similar studies (41). Rb and V, which have potential nutritional benefits (42, 43) and Li, which has played an especially important role during the early fetal development (44, 45) was included in the conditional trace elements group. PM_2.5_ was treated as a separate group. Additionally, based on the four-group analysis, three mixed group was created to explore interaction terms between PM_2.5_ (external exposure) and metal levels (internal exposure), examining potential synergistic effects. This approach has been employed in the research of scenarios (46).

WQS, GWQS and BKMR were performed using the “gWQS”, “group WQS” and “bkmr” package in R (version 4.3.3). All statistical tests were conducted with significance determined by two-tailed *P* < 0.05.

## 3 Results

### 3.1 Population characteristics

The demographic and pregnancy-related characteristics of the 235 participants are summarized in Table 1. Women with PTB had significantly shorter gestational durations, and delivered infants with lower birth weights and lengths compared with term controls. Other maternal characteristics did not differ significantly between the two groups. Among the 18 maternal blood trace elements (Supplementary Table S1), significant group differences were observed for Ag, Cd, Li, Se, Ni, and Zn. Supplementary Table S2 highlights differences in PM_2.5_ exposure, with higher levels observed in the PTB group during the second trimester and from early to mid-pregnancy. Correlation coefficients among pollutants ranged from 0.00 to 0.88 (Supplementary Figure S1).

**Table 1:** Basic characteristics of PTB and controls.

**Characteristics**	**PTB (*N* = 111)**	**Control (*N* = 124)**	***P*-value**
**Maternal age (years)**			0.260
< 25	5 (4.50)	6 (4.84)	
25–29	45 (40.54)	58 (46.77)	
30–35	44 (39.64)	45 (36.29)	
≥35	17 (15.32)	15 (12.10)	
Mean ± SD	30.39 ± 3.65	30.07 ± 3.74	0.515
**Prepregnancy BMI (kg/m** ^2^ **)**			0.038
Underweight (< 18.50)	13 (11.71)	15 (12.10)	
Normal weight (18.50–24.99)	75 (67.57)	85 (68.55)	
Overweight (25–27.99)	16 (14.41)	17 (13.71)	
Obesity (≥28.00)	7 (6.31)	7 (5.65)	
Mean ± SD	22.92 ± 5.92	21.77 ± 4.22	0.203
**Education**			0.918
High school or less	17 (15.32)	15 (12.10)	
Some college	42 (37.84)	37 (29.84)	
College graduate	41 (36.94)	65 (52.42)	
Graduate degree or higher	11 (9.91)	7 (5.65)	
**Occupation**			0.924
Yes	85 (76.58)	98 (79.03)	
No	26 (23.42)	26 (20.97)	
**Family income (CNY)**			0.460
< 60000	32 (28.83)	43 (34.68)	
60000–99999	31 (27.93)	25 (20.16)	
100000–199999	36 (32.43)	39 (31.45)	
>200000	12 (10.81)	17 (13.71)	
**Prior pregnancies**			0.696
0	61 (54.95)	59 (47.58)	
≥1	50 (45.05)	65 (52.42)	
**ART**			0.619
Yes	12 (10.81)	10 (8.06)	
No	99 (89.19)	114 (91.94)	
**Parity**			0.940
Multiparous	24 (21.62)	36 (29.03)	
Primiparous	87 (78.38)	88 (70.97)	
**Drinking**			1.000
Yes	103 (92.79)	116 (93.55)	
No	8 (7.21)	8 (6.45)	
**Smoking**			1.000
Yes	108 (97.30)	121 (97.58)	
No	3 (2.70)	3 (2.42)	
**Delivery mode**			0.182
Natural labor	50 (46.73)	71 (55.47)	
Cesarean section	57 (53.27)	57 (44.73)	
**Fetal gender**			0.470
Male	66 (61.68)	73 (57.03)	
Female	41 (38.32)	55 (42.97)	
Gestation length (week)	35.03 ± 1.66	38.99 ± 1.01	< 0.001
Fetal_weight (g)	2,560.99 ± 513.05	3,345.65 ± 369.40	< 0.001
Fetal_height (cm)	46.35 ± 3.11	49.78 ± 1.76	< 0.001

Notes: Categorical variables were analyzed using Chi-square test (Fisher's exact test for small counts, *n* < 5). Continuous variables were analyzed using independent t-test and are presented as mean ± SD. All tests are two-sided; *P* < 0.05 was considered significant.

### 3.2 Associations of Individual Exposures with PTB

**Figure 1:**
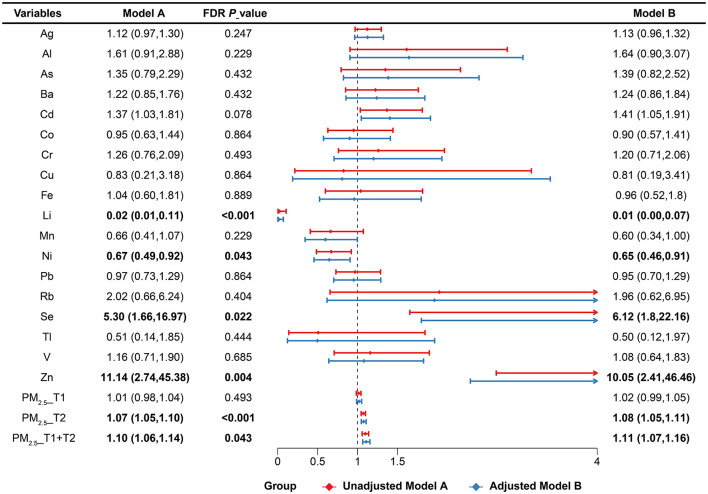
The association between maternal plasma heavy metals and PM_2.5_ concentration with PTB risk estimated by logistic regression models. FDR *P*-values from the unadjusted logistic regression models were adjusted for multiple comparisons using the Benjamini–Hochberg False Discovery Rate (FDR) method. Crude model was expressed by crude odds ratio (95% confidence interval). Single-mixture model was adjusted according to the occupational status, ART, parity, pregnancy history, education, income, mother's age, pre-pregnancy BMI and mode of labor. Abbreviations: Ag, silver; Al, aluminum; As, arsenic; Ba, barium; Cd, cadmium; Co, cobalt; Cr, chromium; Cu, copper; Fe, iron; Li, lithium; Mn, manganese; Ni, nickel; Pb, lead; Rb, rubidium; Se, selenium; Tl, thallium; V, vanadium; Zn, zinc; PM_2.5__T1, fine particulate matter < 2.5 μm at first trimester; PM_2.5__T2, fine particulate matter < 2.5 μm at second trimester; PM_2.5__T1 + T2, cumulative fine particulate matter < 2.5 μm across early-to-mid trimester.

Logistic regression identified several significant associations (Figure 1). In the Unadjusted model, Cd (OR: 1.37, 95% CI: 1.03, 1.81), Se (OR: 5.30, 95% CI: 1.66, 16.97), and Zn (OR: 11.14, 95% CI: 2.74, 45.38) were inversely associated with PTB, while Li (OR: 0.02, 95% CI: 0.01, 0.11) and Ni (OR: 0.67, 95% CI: 0.49, 0.92) showed inverse associations. PM_2.5_ exposure during the second trimester (OR: 1.07, 95% CI: 1.05, 1.10) and the early-to-mid pregnancy period (OR: 1.10, 95% CI: 1.06, 1.14) was also positively associated with PTB risk. After applying the FDR correction to account for multiple comparisons, Li, Ni, Zn, Se, and PM_2.5_ in the second trimester remained statistically significant, while other exposures were no longer significant. These associations remained robust after adjustment for occupational status, ART, parity, pregnancy history, education, income, mother's age, pre-pregnancy BMI and mode of delivery in the single-mixture model. Dose-response curves further suggested nonlinear associations for Ag, Cd, Cr, Se, Zn, and PM_2.5_ during multiple pregnancy windows (Supplementary Figure S2; *P* for nonlinearity < 0.05).

### 3.3 Associations of exposure mixtures with PTB

**Figure 2:**
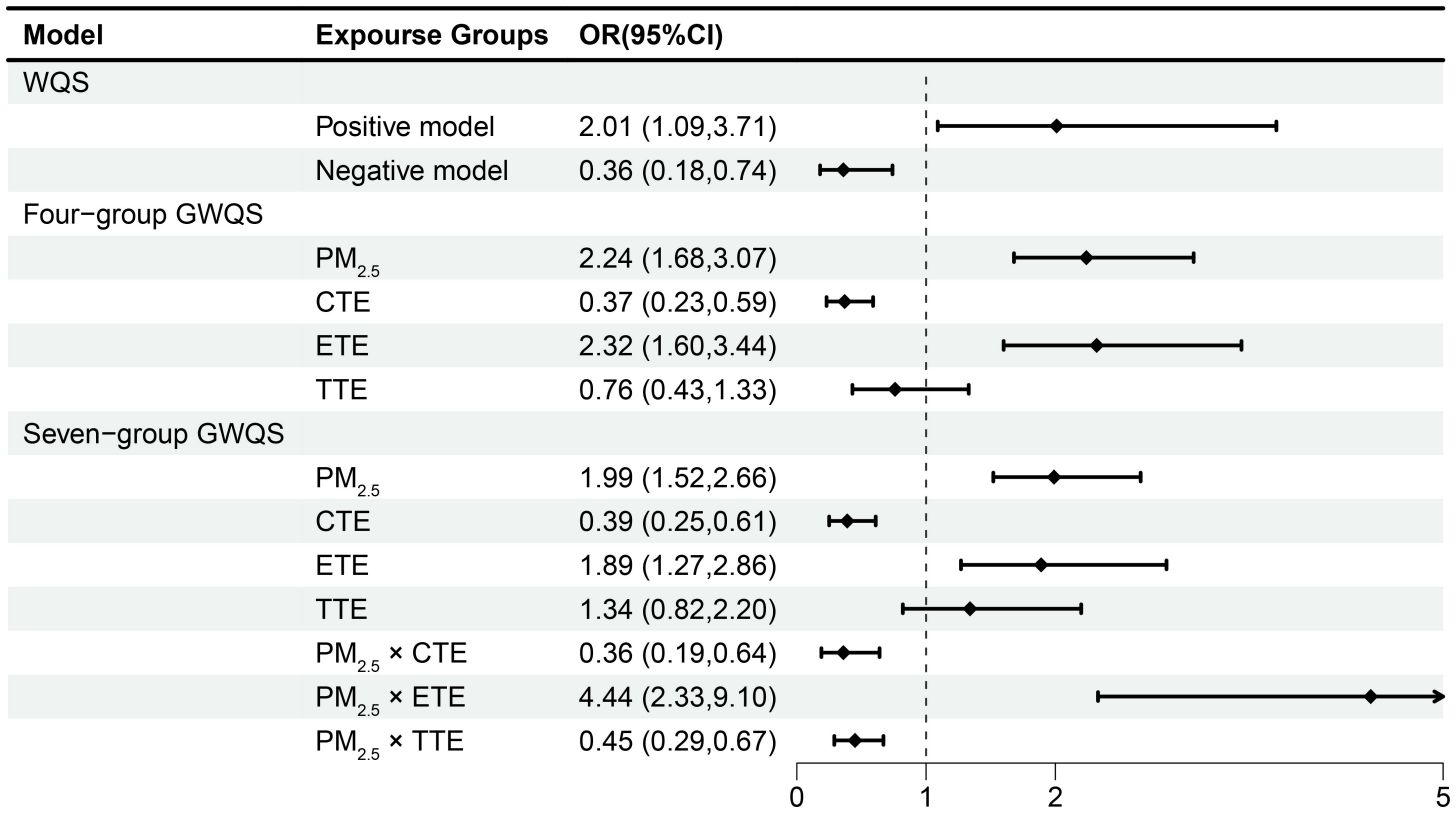
Odds ratios (ORs) and 95% confidence intervals for PTB associated with PM_2.5_ and heavy metal mixtures from weighted quantile sum (WQS) and grouped WQS (GWQS) models. The four-group GWQS included PM_2.5_, conditionally essential elements (CTE), essential trace elements (ETE), and toxic trace elements (TTE). The seven-group GWQS additionally modeled interactions between PM_2.5_ and each trace element group. The dashed vertical line indicates the null value (OR = 1). Models were adjusted according to the occupational status, ART, parity, pregnancy history, education, income, mother's age, pre-pregnancy BMI and mode of labor. Abbreviations: PM_2.5_, fine particulate matter < 2.5 μm; CTE, conditionally essential trace elements; ETE, essential trace elements; TTE, toxic trace elements.

**Figure 3:**
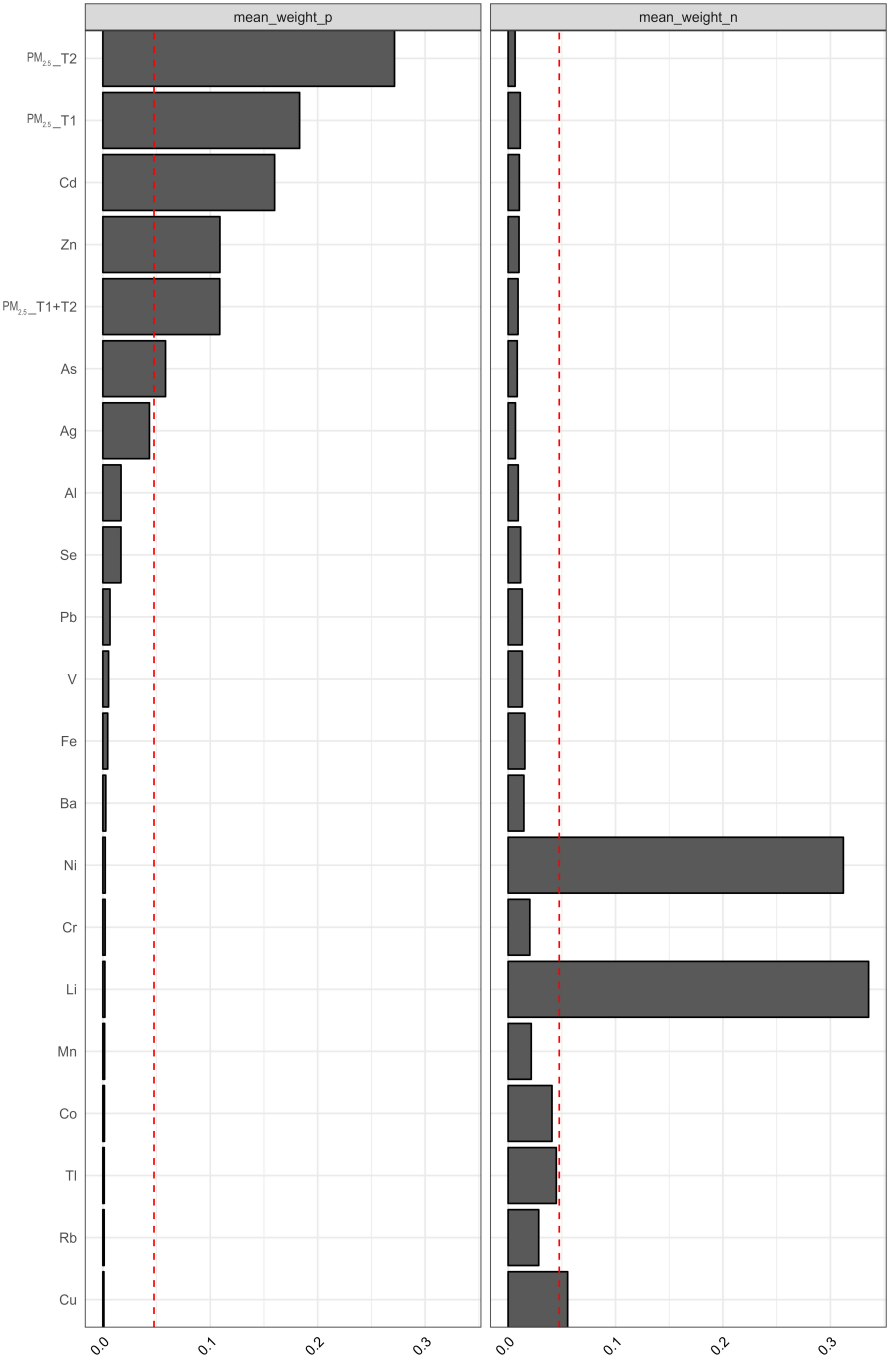
Mean weights of individual exposures derived from WQS regression models assessing the joint effects of PM_2.5_ and heavy metal mixtures on PTB risk. Abbreviations: Ag, silver; Al, aluminum; As, arsenic; Ba, barium; Cd, cadmium; Co, cobalt; Cr, chromium; Cu, copper; Fe, iron; Li, lithium; Mn, manganese; Ni, nickel; Pb, lead; Rb, rubidium; Se, selenium; Tl, thallium; V, vanadium; Zn, zinc; PM_2.5__T1, fine particulate matter < 2.5 μm at first trimester; PM_2.5__T2, fine particulate matter < 2.5 μm at second trimester; PM_2.5__T1 + T2, cumulative fine particulate matter < 2.5 μm across early-to-mid trimester.

In the conventional WQS model (Figure 2), a significant positive association was observed between the exposure mixture and PTB (OR: 2.01, 95% CI: 1.09, 3.71), with PM_2.5_ exposure during the second trimester contributing the largest weight to the mixture index. In contrast, the negative model showed a significant inverse association (OR: 0.36, 95% CI: 0.18, 0.74), in which Li had the highest weight, followed by Ni. These weights reflect the relative contribution of each metal within the mixture, rather than their independent effects (Figure 3).

**Figure 4:**
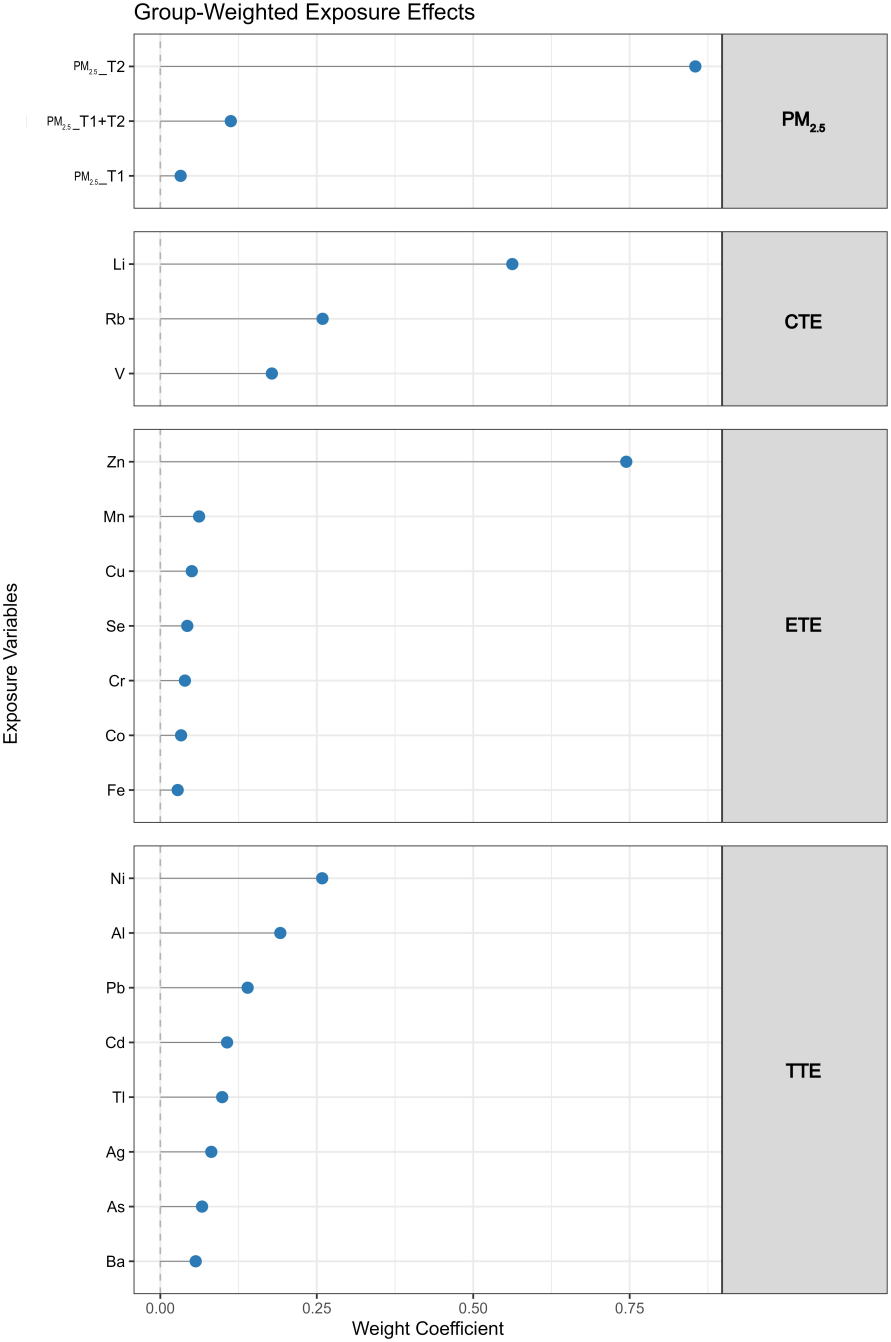
Group-weighted contributions of PM_2.5_ and heavy metals to overall mixture effects on PTB. Abbreviations: Ag, silver; Al, aluminum; As, arsenic; Ba, barium; Cd, cadmium; Co, cobalt; Cr, chromium; Cu, copper; Fe, iron; Li, lithium; Mn, manganese; Ni, nickel; Pb, lead; Rb, rubidium; Se, selenium; Tl, thallium; V, vanadium; Zn, zinc; PM_2.5__T1, fine particulate matter < 2.5 μm at first trimester; PM_2.5__T2, fine particulate matter < 2.5 μm at second trimester; PM_2.5__T1 + T2, cumulative fine particulate matter < 2.5 μm across early-to-mid trimester; PM_2.5_, fine particulate matter < 2.5 μm; CTE, conditionally essential trace elements; ETE, essential trace elements; TTE, toxic trace elements.

When the mixture was divided into four exposure groups—PM_2.5_, conditionally essential trace elements (CTE), essential trace elements (ETE), and toxic trace elements (TTE)—Four-group GWQS regression analysis showed that both PM_2.5_ (OR: 2.24, 95% CI: 1.68, 3.07) and ETE (OR: 2.32, 95% CI: 1.60, 3.44) were positively associated with PTB, with second-trimester PM_2.5_ and zinc (Zn) contributing the most to their respective group indices (Figure 2). In contrast, CTE was negatively associated with PTB (OR: 0.37, 95% CI: 0.23, 0.59), with Li showing the highest weight, while TTE was not significantly associated with PTB (OR: 0.76, 95% CI: 0.43, 1.33) (Figure 4).

The extended seven-group GWQS model further explored the interaction effects between PM_2.5_ and each trace element group (Figure 2). PM_2.5_ remained positively associated with increased risk of PTB (OR: 1.99, 95% CI: 1.52, 2.66), while CTE was associated with reduced risk (OR: 0.39, 95% CI: 0.25, 0.61), and ETE was associated with increased risk (OR: 1.89, 95% CI: 1.27, 2.86). No significant association was observed for TTE (OR: 1.34, 95% CI: 0.82, 2.20). The relative contribution of each group remained consistent with the results from the four-group GWQS model. Furthermore, the inclusion of interaction terms indicated that the co-exposure of PM_2.5_ and ETE was associated with a higher PTB risk (OR: 4.44, 95% CI: 2.33, 9.10), whereas the association between PM_2.5_ and PTB showed weaker associations with higher CTE (OR: 0.36, 95% CI: 0.29, 0.67) and TTE (OR: 0.45, 95% CI: 0.29, 0.67) (Supplementary Figure S3).

**Figure 5:**
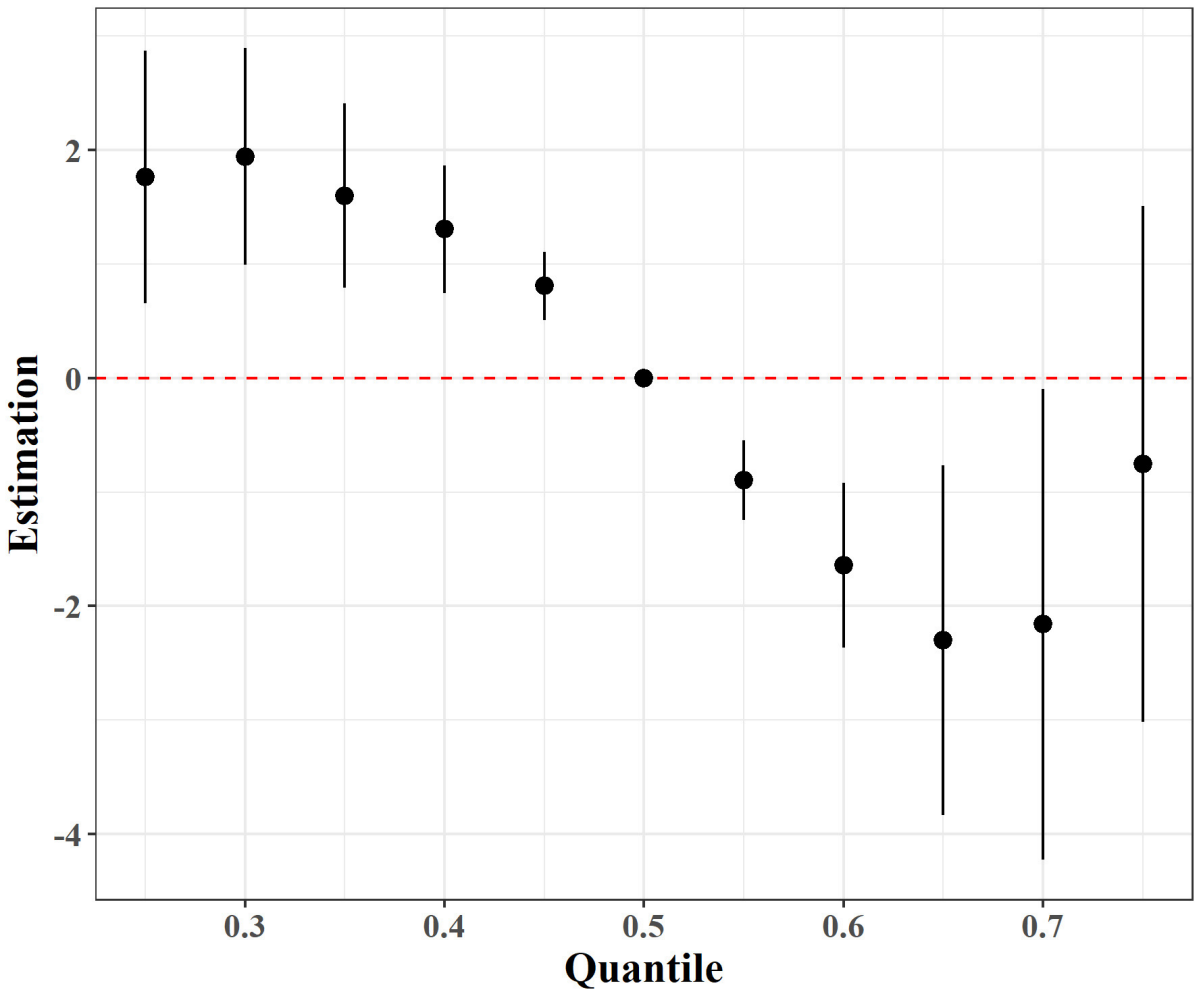
Overall exposure–response function of the PM_2.5_ and heavy metal mixtures estimated by BKMR.

**Figure 6:**
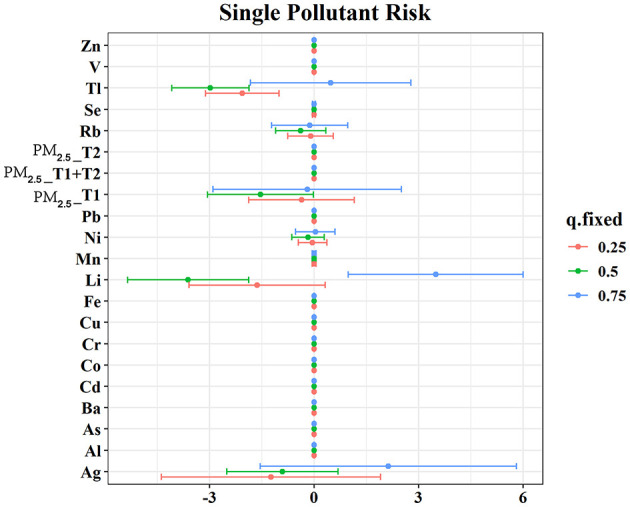
Univariate exposure–response functions for PM_2.5_ and heavy metal mixtures estimated by BKMR. Each curve shows the estimated change in PTB risk with one exposure varying across its range while holding other exposures at their median values. Abbreviations: Ag, silver; Al, aluminum; As, arsenic; Ba, barium; Cd, cadmium; Co, cobalt; Cr, chromium; Cu, copper; Fe, iron; Li, lithium; Mn, manganese; Ni, nickel; Pb, lead; Rb, rubidium; Se, selenium; Tl, thallium; V, vanadium; Zn, zinc; PM_2.5__T1, fine particulate matter < 2.5 μm at first trimester; PM_2.5__T2, fine particulate matter < 2.5 μm at second trimester; PM_2.5__T1 + T2, cumulative fine particulate matter < 2.5 μm across early-to-mid trimester.

In the BKMR analysis, the overall effect of the multi-pollutant mixture on PTB exhibited a nonlinear pattern, with an initial decrease followed by an increase, and the lowest risk observed at the 65th percentile of the exposure-response function (Figure 5). In the single-exposure analysis, Li showed a complex, non-monotonic association with PTB: Li was negatively associated with PTB at the 50th percentile of its distribution but positively associated at the 75th percentile, suggesting potential threshold or saturation effects (Figure 6). To further assess the relative contribution of each exposure, Posterior Inclusion Probabilities (PIPs) were calculated from the BKMR model (Supplementary Table S4). Li, Tl, and PM_2.5_ during the first trimester (PM_2.5__T1) exhibited high conditional PIP values (≥0.7), indicating stronger and more consistent associations with PTB. In contrast, most other metals had relatively low PIPs, suggesting weaker associations.

## 4 Discussion

In this nested case-control study, we found that both individual and joint exposures to PM_2.5_ and trace elements during pregnancy were significantly associated with the risk of PTB. Logistic regression models identified Cd, Se, and Zn as positively associated with PTB, whereas Li and Ni showed inversely associations. PM_2.5_ exposure during the second trimester and across early-to-mid pregnancy was also consistently linked to elevated PTB risk. Mixture analyses using WQS and GWQS models further revealed that PM_2.5_ and ETE (particularly Zn) were the dominant contributors to increased risk, while CEE, especially Li, were inversely associated with PTB. However, it should be noted that several trace metals were highly correlated (Supplementary Figure S1). While WQS and BKMR account for collinearity and provide stable mixture estimates, high correlations limit the ability to attribute effects to any single metal. Therefore, WQS weights should be interpreted as reflecting the relative importance of metals within the mixture, rather than independent effects of individual metals.

In addition, the extended GWQS model suggested potential positively interactions between PM_2.5_ and ETE group, and were suggestive of inverse interactions between PM_2.5_ and both CTE and TTE group. Notably, BKMR models identified a nonlinear overall effect of the exposure mixture, with the lowest PTB risk at the 65th percentile of exposure. The association between Li and PTB demonstrated a non-monotonic, suggesting possible threshold or saturation effects. Collectively, these findings highlight both positive and negative associations of specific trace metals and their interactions with PM_2.5_, providing new insights into the complex mixture effects on adverse pregnancy outcomes.

Our findings that have reported positive associations between prenatal exposure to Cd (47) and increased risk of PTB, as well as associations between lower maternal levels of Zn (21) and Se (48) and heightened PTB risk. Cd is a well-established reproductive toxicant that can cross the placental barrier (49) and induce oxidative stress and inflammation (50, 51). It also disrupts cellular redox balance, potentially leading to DNA damage and impaired cell homeostasis (52), These biological effects are strongly implicated in the pathophysiology of PTB. Zn and Se are essential trace elements involved in antioxidant defense, immune regulation, and fetal development. Consistent with prior studies, our results suggest that insufficient levels of Zn and Se during pregnancy may increase the risk of PTB, likely due to impaired antioxidant capacity (53) and placental dysfunction (54).

Consistent with previous studies (55–59), sustained exposure to PM_2.5_ during pregnancy was strongly associated with PTB risk. Proposed biological mechanisms include systemic inflammation, endocrine disruption, and impaired placental development (60, 61). Recent metabolomics evidence further suggests that PM_2.5_ may disrupt protein digestion and the metabolism of aromatic amino acids, such as phenylalanine, tyrosine, and tryptophan, which are essential for fetal growth and immune regulation (62). These findings emphasize the need for targeted interventions to reduce maternal PM_2.5_ exposure.

In contrast, Li and Ni were inversely associated with PTB risk in both single and mixture-exposure models. Lithium exposure, primarily derived from drinking water and diet, varies considerably by region. Although high-dose lithium salts used in psychiatric treatment carry toxicity risks, emerging evidence suggests that low-dose lithium may offer physiological benefits far beyond its established psychiatric applications. A comprehensive review (63) reported that lithium concentrations ≤ 0.5 mM are associated with improvements in cardiovascular, musculoskeletal, metabolic, and cognitive functions, likely through suppression of inflammatory responses and activation of antioxidant pathways, thereby exerting broad systemic protective effects. Emerging evidence also highlights lithium potential protective role in reproductive health. Animal studies have shown that dietary lithium supplementation significantly increases lifetime egg production in female Drosophila melanogaster, potentially via modulation of insulin-like signaling and genes involved in follicular development (64). Epidemiologic data further indicate that higher lithium levels in drinking water are linked to reduced risks of certain cancers (65), suggesting broader health benefits at environmentally relevant exposures. Similarly, Ni (66), traditionally considered a potentially toxic element, has been reported to support certain enzymatic functions at trace levels (67).

However, GWQS models further suggested a potential interaction between PM_2.5_ and essential elements such as zinc, which was associated with higher PTB risk, whereas conditional essential elements like lithium and toxic metals showed patterns indicative of inverse interactions. The nonlinear exposure-response relationship observed in the BKMR analysis, with the lowest PTB risk around the 65th percentile of exposure, underscores the complexity of environmental mixture effects and the importance of considering dose-response thresholds. Overall, these findings indicate that lithium was inversely associated with PTB within the context of environmental pollutant exposure, highlighting its potential role in modulating risk.

This study has several notable strengths. First, the use of a nested case-control design with individual-level biomonitoring data provides a reliable basis for assessing prenatal environmental exposures. Second, we quantified 18 trace elements using ICP-MS and incorporated high-resolution PM_2.5_ estimates, enabling detailed exposure characterization. Third, by applying complementary mixture modeling techniques, including WQS, GWQS, and BKMR, we were able to identify both dominant contributors and complex nonlinear or interaction effects, offering a more nuanced understanding of exposure–outcome association.

However, the study has several limitations. First, the GWQS model requires a priori grouping of exposures, which may lead to exposure misclassification and potentially obscure important contributors within groups. In addition, although we examined multiple trace elements and PM_2.5_, other coexisting environmental pollutants (e.g., gaseous air pollutants, endocrine-disrupting chemicals) were not accounted for, possibly resulting in residual confounding. Second, our study was a nested case-control study rather than a community-based sample. This design may introduce selection bias, as cases and controls were not randomly selected from the general population. Consequently, the study population may not fully represent all pregnant women in the region, which could limit the generalizability of our findings. In addition, as a case-control study, the design allows assessment of associations but does not permit causal inference between environmental mixture exposures and PTB. Future studies, including experimental models or prospective cohorts, are needed to confirm these associations and further explore potential causal mechanisms. Third, the relatively small sample size may reduce statistical power and increase susceptibility to overfitting, particularly in high-dimensional mixture models such as BKMR and GWQS with interaction terms. Moreover, PM_2.5_ exposure was estimated using a high-resolution model based on satellite, which may still not fully capture personal-level variability in exposure.

Future research should address these limitations by conducting multi-center, large-sample prospective cohort studies with repeated measurements of trace metal concentrations across pregnancy and inclusion of additional environmental exposures. Individual-level assessments of PM_2.5_ using personal monitoring devices would further enhance exposure accuracy. In addition, mechanistic and experimental studies are warranted to elucidate the dose-specific roles of conditionally essential elements, such as lithium, and to confirm these associations while exploring potential causal mechanisms.

## 5 Conclusions

In summary, this study provides novel epidemiological evidence that prenatal exposure to PM_2.5_ and multiple trace elements is associated with the risk of PTB. Specifically, Cd, Zn, Se, and PM_2.5_, particularly during the second trimester, were positively associated with PTB risk, whereas Li and Ni showed inverse associations. Multiple mixture modeling approaches revealed notable interaction and nonlinear exposure–response patterns, underscoring the complexity of combined environmental exposures and the limitations of traditional single-exposure analyses. These findings enhance our understanding of how coexposure to air pollution and trace elements may influence pregnancy outcomes. Future multi-center, large-sample prospective cohort studies and mechanistic investigations are warranted to validate these associations, elucidate the underlying biological pathways, and inform evidence-based environmental and public health strategies to reduce PTB risk.

## Data Availability

The data analyzed in this study is subject to the following licenses: The data are not publicly available due to privacy. Requests to access these datasets should be directed to zhouwenzheng@126.com.
