# Supplementary material for: Joint effects of prenatal PM2.5 and heavy metals on preterm birth: evidence from a nested case–control study in China
Source: Front Public Health. 2026 Jan 21;13:1701012. doi: 10.3389/fpubh.2025.1701012 (PMC12869712; doi:10.3389/fpubh.2025.1701012)
Supplement: Supplementary file 1 [file Data_Sheet_1.docx]

***Supplementary Material***

**Table of Contents**

**Supplementary Figure S1.** Correlations of Second-Trimester Maternal plasma Trace Elements with PM_2.5_ exposure in the First Trimester, Second Trimester, and First Through Second Trimesters.

**Supplementary Figure S2.** Dose−response relationships of metal and PM_2.5_ with PTB during pregnancy fitted with restricted cubic spline (RCS) models.

**Supplementary Figure S3.** Seven-group WQS regression analysis of PM2.5-metal mixtures and PTB risk.

**Supplementary Table S1.** The concentration of 18 elements in maternal blood (μg/L).

**Supplementary Table S2.** PM_2.5_ Exposure during Different Gestational Stages of PTB and controls (μg/m³).

**Supplementary Table S3.** Association between metal and PM_2.5_ concentration with PTB risk.

**Supplementary Table S4.** Posterior Inclusion Probabilities (PIPa) of Exposures from the BKMR Model.

**
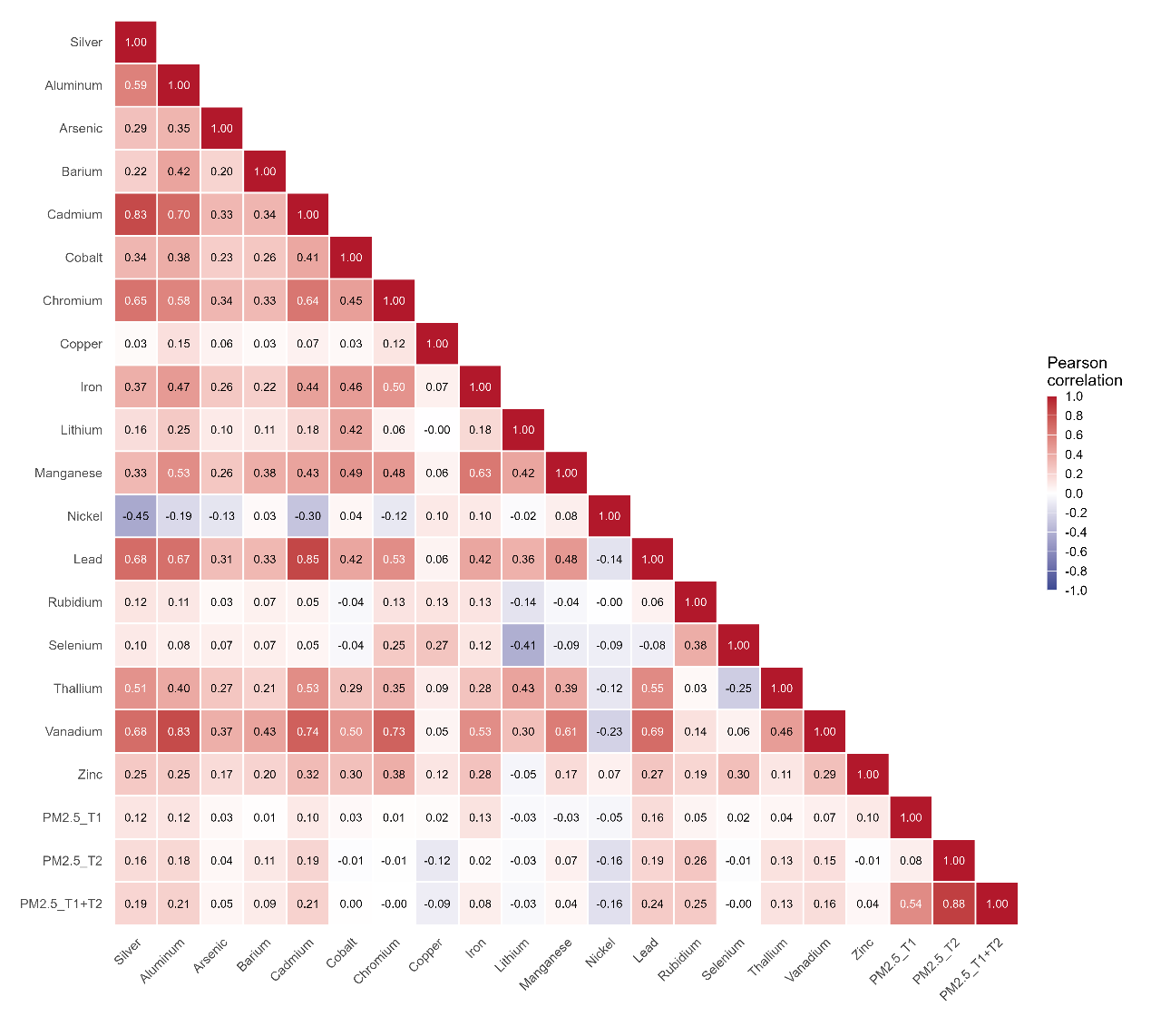
**

**Supplementary Figure1.** Correlations of Second-Trimester Maternal plasma Trace Elements with PM_2.5_ exposure in the First Trimester, Second Trimester, and First Through Second Trimesters.

Abbreviations: PM_2.5__T1, fine particulate matter <2.5 μm at first trimester; PM_2.5__T2, fine particulate matter <2.5 μm at second trimester; PM_2.5__T1+T2, cumulative fine particulate matter <2.5 μm across first to middle trimester.


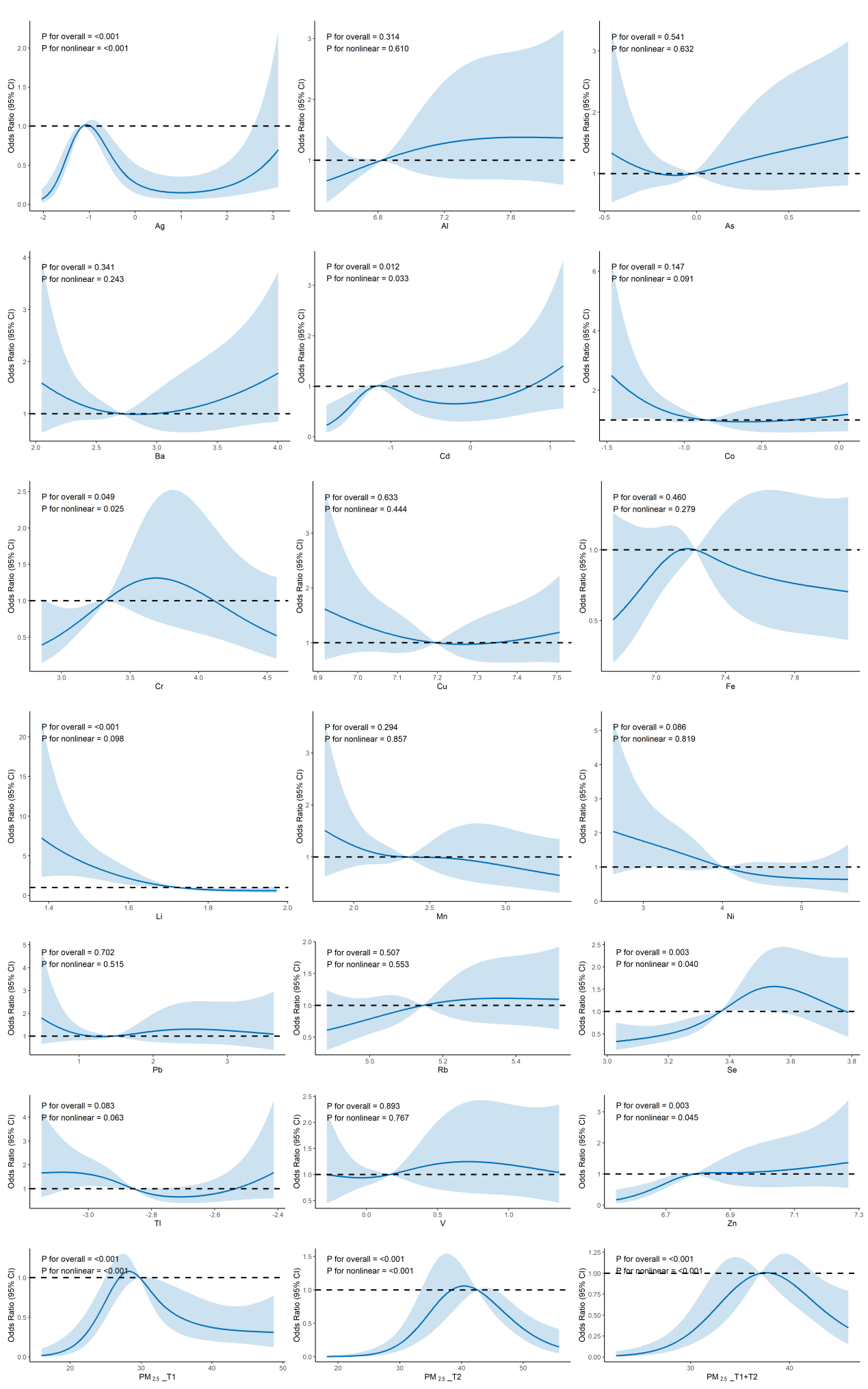


**Supplementary Figure S2.** Dose−response relationships of metal and PM_2.5_ with PTB during pregnancy fitted with restricted cubic spline (RCS) models. RCS models were adjusted for all the covariates including occupational status, ART, parity, pregnancy history, education, income, mother’s age, pre-pregnancy BMI and mode of delivery.

Abbreviations: Ag, silver; Al, aluminum; As, arsenic; Ba, barium; Cd, cadmium; Co, cobalt; Cr, chromium; Cu, copper; Fe, iron; Li, lithium; Mn, manganese; Ni, nickel; Pb, lead; Rb, rubidium; Se, selenium; Tl, thallium; V, vanadium; Zn, zinc; PM_2.5__T1, fine particulate matter <2.5 μm at first trimester; PM_2.5__T2, fine particulate matter <2.5 μm at second trimester; PM_2.5__T1+T2, cumulative fine particulate matter <2.5 μm across first to middle trimester.


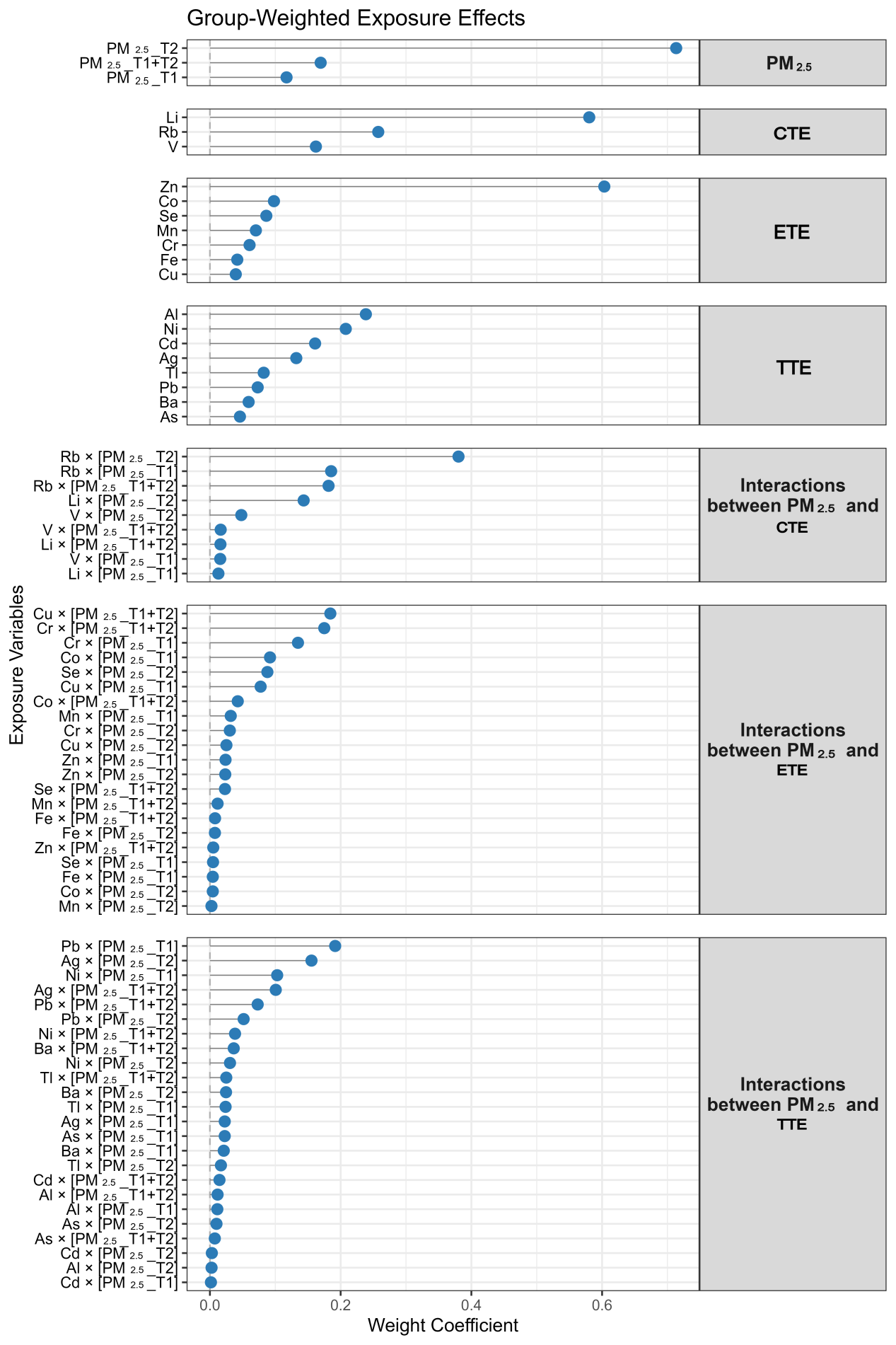


**Supplementary Figure S3** Seven-group WQS regression analysis of PM_2.5_-metal mixtures and PTB risk.

Abbreviations: Ag, silver; Al, aluminum; As, arsenic; Ba, barium; Cd, cadmium; Co, cobalt; Cr, chromium; Cu, copper; Fe, iron; Li, lithium; Mn, manganese; Ni, nickel; Pb, lead; Rb, rubidium; Se, selenium; Tl, thallium; V, vanadium; Zn, zinc; PM_2.5__T1, fine particulate matter <2.5 μm at first trimester; PM_2.5__T2, fine particulate matter <2.5 μm at second trimester; PM_2.5__T1+T2, cumulative fine particulate matter <2.5 μm across first to middle trimester, PM_2.5_, fine particulate matter <2.5 μm; CTE, conditionally essential trace elements; ETE, essential trace elements; TTE, toxic trace elements.

**Supplementary Table S1.** The concentration of 18 elements in maternal blood (μg/L).

| Trace Elements | PTB (N=111) | | Control (N=124) | | LOD(μg / L ) | Detection rate (%) | *P*_value |
| --- | --- | --- | --- | --- | --- | --- | --- |
|  | Mean ± SD | Median (25th, 75th) | Mean ± SD | Median (25th, 75th) |  |  |  |
| Ag | 2.04 ± 3.30 | 0.28 (0.15, 3.32) | 27.32 ± 160.79 | 0.38 (0.21, 2.17) | 0.0003 | 100 | 0.012* |
| Al | 1136.42 ± 787.6 | 880.97 (734.68, 1185.30) | 1269.7 ± 983.71 | 929.51 (768.88, 1455.71) | 0.3473 | 100 | 0.053 |
| As | 1.14 ± 0.65 | 0.96 (0.79, 1.25) | 2.01 ± 6.87 | 1.02 (0.79, 1.33) | 0.0012 | 100 | 0.619 |
| Ba | 19.93 ± 15.18 | 14.53 (10.74, 25.69) | 48.83 ± 215.92 | 15.04 (10.33, 26.82) | 0.0149 | 100 | 0.790 |
| Cd | 0.64 ± 0.82 | 0.29 (0.21, 0.64) | 0.93 ± 1.27 | 0.31 (0.26, 0.94) | 0.001 | 100 | 0.009* |
| Co | 0.51 ± 0.29 | 0.44 (0.32, 0.60) | 1.03 ± 4.38 | 0.4 (0.3, 0.57) | 0.0003 | 100 | 0.171 |
| Cr | 37.71 ± 28.87 | 26.41 (20.46, 39.89) | 37.61 ± 20.83 | 28.57 (23.11, 47.35) | 0.0062 | 100 | 0.108 |
| Cu | 1369.58 ± 254.26 | 1334.76 (1221.25, 1480.62) | 1370.98 ± 338.95 | 1331.04 (1168.94, 1493.55) | 0.3015 | 100 | 0.596 |
| Fe | 1802.28 ± 2026.61 | 1371.83 (1180.40, 1851.59) | 1858.93 ± 2979.96 | 1384.82 (1190.76, 1686.46) | 0.0469 | 100 | 0.904 |
| Li | 5.94 ± 1.14 | 5.99 (5.28, 6.53) | 5.22 ± 1.14 | 4.92 (4.36, 6.05) | 0.0176 | 100 | <0.001* |
| Mn | 16.94 ± 28.08 | 11.49 (7.91, 16.48) | 12.77 ± 10.73 | 10.15 (7.67, 14.78) | 0.0032 | 100 | 0.136 |
| Ni | 89.38 ± 75.88 | 62.49 (39.39, 117.21) | 70.25 ± 70.53 | 48.76 (26.46, 78.89) | 0.0093 | 100 | 0.008* |
| Pb | 8.39 ± 9.92 | 4.27 (2.56, 10.37) | 8.51 ± 10.82 | 4.25 (2.47, 9.38) | 0.0055 | 100 | 0.711 |
| Rb | 176.95 ± 60.88 | 168.83 (151.65, 190.43) | 183.75 ± 60.9 | 174.2 (153.18, 195.76) | 0.0017 | 100 | 0.275 |
| Se | 28.96 ± 7.8 | 27.09 (23.71, 32.61) | 31.27 ± 6.83 | 31.21 (26.44, 34.28) | 0.0143 | 100 | 0.002* |
| Tl | 0.06 ± 0.01 | 0.06 (0.05, 0.07) | 0.06 ± 0.01 | 0.06 (0.05, 0.06) | 0.0002 | 100 | 0.115 |
| V | 1.61 ± 1.47 | 1.17 (0.91, 1.64) | 1.61 ± 1.01 | 1.2 (0.92, 2.03) | 0.0024 | 100 | 0.490 |
| Zn | 900.87 ± 210.31 | 851.39 (765.48, 981.02) | 988.43 ± 217.98 | 922.86 (846.68, 1083.1) | 0.0521 | 100 | <0.001* |

Abbreviations: SD, standard deviation; LOD, limit of detection; Ag, silver; Al, aluminum; As, arsenic; Ba, barium; Cd, cadmium; Co, cobalt; Cr, chromium; Cu, copper; Fe, iron; Li, lithium; Mn, manganese; Ni, nickel; Pb, lead; Rb, rubidium; Se, selenium; Tl, thallium; V, vanadium; Zn, zinc; PM_2.5__T1, fine particulate matter <2.5 μm at first trimester; PM_2.5__T2, fine particulate matter <2.5 μm at second trimester; PM_2.5__T1+T2, cumulative fine particulate matter <2.5 μm across first to middle trimester.

A p-value were derived from Student`s t-test or Mann–Whitney U tests; with *, p-value < 0.05.

**Supplementary Table S2.** PM_2.5_ Exposure during Different Gestational Stages of PTB and controls (μg/m³).

| PM_2.5_ | PTB (N=111)  mean ± SD | Control (N=124)  mean ± SD | *P*_value |
| --- | --- | --- | --- |
| PM_2.5__T1 | 30.68 ± 10.47 | 31.68 ± 6.99 | 0.310 |
| PM_2.5__T2 | 34.83 ± 13.85 | 43.27 ± 7.13 | <0.001^*^ |
| PM_2.5__T1+T2 | 33.06 ± 9.13 | 38.31 ± 5.48 | <0.001^*^ |

PM_2.5__T1, fine particulate matter <2.5 μm at first trimester; PM_2.5__T2, fine particulate matter <2.5 μm at second trimester; PM_2.5__T1+T2, cumulative fine particulate matter <2.5 μm across first to middle trimester.

A p-value were derived from Student`s t-test; with *, p-value < 0.05.

**Supplementary Table S3.** Association between metal and PM_2.5_ concentration with PTB risk.

| Mixture  concentration | OR (95%CI) | OR (95%CI) | FDR *P*_value^c^ |
| --- | --- | --- | --- |
|  | Unadjusted Model^a^ | Adjusted Model^b^ |  |
| Ag | 1.12 (0.97,1.30) | 1.13 (0.96,1.32) | 0.247 |
| Al | 1.61 (0.91,2.88) | 1.64 (0.90,3.07) | 0.229 |
| As | 1.35 (0.79,2.29) | 1.39 (0.82,2.52) | 0.432 |
| Ba | 1.22 (0.85,1.76) | 1.24 (0.86,1.84) | 0.432 |
| Cd | 1.37 (1.03,1.81) | 1.41 (1.05,1.91) | 0.078 |
| Co | 0.95 (0.63,1.44) | 0.90 (0.57,1.41) | 0.864 |
| Cr | 1.26 (0.76,2.09) | 1.20 (0.71,2.06) | 0.493 |
| Cu | 0.83 (0.21,3.18) | 0.81 (0.19,3.41) | 0.864 |
| Fe | 1.04 (0.60,1.81) | 0.96 (0.52,1.80 | 0.889 |
| Li | **0.02 (0.01,0.11)** | **0.01 (0.00,0.07)** | **<0.001** |
| Mn | 0.66 (0.41,1.07) | 0.60 (0.34,1.00) | 0.229 |
| Ni | **0.67 (0.49,0.92)** | **0.65 (0.46,0.91)** | **0.043** |
| Pb | 0.97 (0.73,1.29) | 0.95 (0.70,1.29) | 0.864 |
| Rb | 2.02 (0.66,6.24) | 1.96 (0.62,6.95) | 0.404 |
| Se | **5.30 (1.66,16.97)** | **6.12 (1.8,22.16)** | **0.022** |
| Tl | 0.51 (0.14,1.85) | 0.50 (0.12,1.97) | 0.444 |
| V | 1.16 (0.71,1.90) | 1.08 (0.64,1.83) | 0.685 |
| Zn | **11.14 (2.74,45.38)** | **10.05 (2.41,46.46)** | **0.004** |
| PM_2.5__T1 | 1.01 (0.98,1.04) | 1.02 (0.99,1.05) | 0.493 |
| PM_2.5__T2 | **1.07 (1.05,1.10)** | **1.08 (1.05,1.11)** | **<0.001** |
| PM_2.5__T1+T2 | **1.10 (1.06,1.14)** | **1.11 (1.07,1.16)** | **0.043** |

^a^ Unadjusted model was expressed by crude odds ratio (95% confidence interval).

^b^ Unadjusted model was adjusted according to the mother’s age, educational level, pre-pregnancy BMI, delivery mode, parity, Prior pregnancies, fetal sex, and family income.

^c^ FDR *P*_values from the unadjusted logistic regression models adjusted for multiple comparisons using the Benjamini–Hochberg False Discovery Rate (FDR) method.

OR and 95% CIs in bold indicate significance at *P* < 0.05.

Abbreviations: Ag, silver; Al, aluminum; As, arsenic; Ba, barium; Cd, cadmium; Co, cobalt; Cr, chromium; Cu, copper; Fe, iron; Li, lithium; Mn, manganese; Ni, nickel; Pb, lead; Rb, rubidium; Se, selenium; Tl, thallium; V, vanadium; Zn, zinc; PM_2.5__T1, fine particulate matter <2.5 μm at first trimester; PM_2.5__T2, fine particulate matter <2.5 μm at second trimester; PM_2.5__T1+T2, cumulative fine particulate matter <2.5 μm across first to middle trimester.

**Supplementary Table S4.** Posterior Inclusion Probabilities (PIP^a^) of Exposures from the BKMR Model.

| Variable | Group^b^ | groupPIP^c^ | condPIP^d^ |
| --- | --- | --- | --- |
| Ag | 1 | 1.00 | 1.00 |
| Al | 1 | 1.00 | 0.00 |
| As | 1 | 1.00 | 0.00 |
| Ba | 1 | 1.00 | 0.00 |
| Cd | 1 | 1.00 | 0.00 |
| Co | 1 | 1.00 | 0.00 |
| Cr | 1 | 1.00 | 0.00 |
| Cu | 1 | 1.00 | 0.00 |
| Fe | 2 | 0.99 | 0.00 |
| Li | 2 | 0.99 | 0.73 |
| Mn | 2 | 0.99 | 0.00 |
| Ni | 2 | 0.99 | 0.15 |
| Pb | 2 | 0.99 | 0.00 |
| Rb | 2 | 0.99 | 0.12 |
| Se | 2 | 0.99 | 0.00 |
| Tl | 3 | 1.00 | 1.00 |
| V | 3 | 1.00 | 0.00 |
| Zn | 3 | 1.00 | 0.00 |
| PM_2.5__T1 | 4 | 1.00 | 1.00 |
| PM_2.5__T2 | 4 | 1.00 | 0.00 |
| PM_2.5__T1+T2 | 4 | 1.00 | 0.00 |

^a^ PIP values range from 0 to 1; higher values indicate stronger evidence that the exposure or group contributes to the outcome.

^b^ Group: Predefined exposure groups used in BKMR.

^c^ Group PIP: Posterior inclusion probability of the exposure group (probability that at least one component in the group is associated with the outcome).

^d^ Conditional PIP: Posterior inclusion probability of the individual exposure conditional on other exposures.

Abbreviations: Ag, silver; Al, aluminum; As, arsenic; Ba, barium; Cd, cadmium; Co, cobalt; Cr, chromium; Cu, copper; Fe, iron; Li, lithium; Mn, manganese; Ni, nickel; Pb, lead; Rb, rubidium; Se, selenium; Tl, thallium; V, vanadium; Zn, zinc; PM_2.5__T1, fine particulate matter <2.5 μm at first trimester; PM_2.5__T2, fine particulate matter <2.5 μm at second trimester; PM_2.5__T1+T2, cumulative fine particulate matter <2.5 μm across first to middle trimester.
